# Arkansas Whetstone

**Published:** 1873-02

**Authors:** 


### Arkansas Whetstone.

A correspondent of the New York *Evening Post* gives the following interesting account of a visit to the famous whetstone quarries of Arkansas:

I paid a visit to the whetstone quarries, about a mile and a half from the village of Hot Springs yesterday. The stone is of extremely fine quality, and is superior to that found near Constantinople, the quartz seems being more infrequent. This whetstone rock is novaculite, and is one of the finest deposits known. The rock crops out on the side of the hill. The vein is worked ten or fifteen feet wide, and is in a coarser variety of the same stone. According to Professor Owen, formerly State geologist, it belongs to the millstone grit, and was once a simple sandstone, and by the permeation of heated alkaline silicous waters has been altered into nearly chemically pure silica. It resembles the finest quality of, and is even superior in whiteness to Carrara marble. From their physical appearance they can hardly be distinguished. The dip of the vein is about forty-two degrees. The quartz seams vary in distance apart generally about six inches, sometimes a foot. They vary in thickness from a hair to half an inch. The quarry was first worked by the present owner, Mr. Barnes, in 1843. He says that the quality is better as he descends, and that the siliceous seams widen as they approach the surface. Large quantities are shipped to New York. The finer varieties are used by engravers, while the coarser are used for bench tools. It is sold here for about one dollar a pound, finished up for use. The workings are very picturesque, branches being laid across the vein to keep off the sun; the rocks are stained very prettily in some places by iron.

The view from the quarries is very fine, and six miles off, the old Outchita quarries, of a similar but coarser stone, can be seen touched by the glow of the evening sun. These works are now abandoned. The deposit worked by Mr. Barnes seems to be inexhaustible.

